# Low glucose and serum levels cause an increased inflammatory factor in 3T3-L1 cell through Akt, MAPKs and NF-кB activation

**DOI:** 10.1080/21623945.2021.1914420

**Published:** 2021-04-25

**Authors:** Hirona Kugo, Wanida Sukketsiri, Kazuko Iwamoto, Satoki Suihara, Tatsuya Moriyama, Nobuhiro Zaima

**Affiliations:** a Department of Applied Biological Chemistry, Graduate School of Agriculture, Kindai University, Nara City, Japan; b Department of Pharmacology, Division of Health and Applied Sciences, Faculty of Science, Prince of Songkla University, Songkhla, Thailand; c Department of Health and Nutrition, Faculty of Health Science, Osaka Aoyama University, Minoh City, Japan; d Agricultural Technology and Innovation Research Institute, Kindai University, Nara, Japan

**Keywords:** Adipocyte, hypoperfusion, reactive oxygen species, TNF-α, Il-1β

## Abstract

Abdominal aortic aneurysm (AAA) involves the degradation of vascular fibres, and dilation and rupture of the abdominal aorta. Hypoperfusion in the vascular walls due to stenosis of the vasa vasorum is reportedly a cause of AAA onset and involves the induction of adventitial ectopic adipocytes. Recent studies have reported that ectopic adipocytes are associated with AAA rupture in both human and hypoperfusion-induced animal models, highlighting the pathological importance of hypoperfusion and adipocytes in AAA. However, the relationship between hypoperfusion and AAA remains unknown. In this study, we investigated the changes in inflammation-related factors in adipocytes at low glucose and serum levels. Low glucose and serum levels enhanced the production of AAA-related factors in 3T3-L1 cells. Low glucose and serum levels increased the activation of protein kinase B (also known as Akt), extracellular signal-regulated protein kinase 1/2, p38, c-Jun N-terminal kinase, and nuclear factor (NF) кB at the protein level. The inflammatory factors and related signalling pathways were markedly decreased following the return of the cells to normal culture conditions. These data suggest that low glucose and serum levels increase the levels of inflammatory factors through the activation of Akt, mitogen activated protein kinase, and NF-κB signalling pathways.

## Introduction

The pathophysiology of abdominal aortic aneurysm (AAA) is characterized by the degradation of the vascular structure due to chronic inflammation and aortic dilation [1]. AAA is often called a silent killer because it has almost no clinical symptoms and the resulting vascular rupture is associated with high mortality [2]. Currently, there is no medical treatment to prevent the dilation and rupture associated with AAA [3]. We previously reported that hypoperfusion in the vascular wall of the vasa vasorum (VV) due to stenosis leads to AAA formation with adventitial ectopic adipocytes [4,5]. Several recent reports have suggested that increased ectopic adipocytes in the vascular wall are associated with AAA development and rupture in both human and experimental animal models [6–10].

VV penetrates the outer layer of the tunica media of larger vessels and supplies blood and nutrition. VV stenosis causes low nutritional supply to the adventitia and outer media of the parent vessel. Under healthy conditions, the adipocytes function normally [11]. Adipose tissue or adipocytes are exposed to various stresses in AAA, including inflammation, hypoxia, and endoplasmic reticulum stress [12,13]. Under stress conditions, the differentiation of adipocytes is inadequate to generate sufficient new adipocytes that results in adipocyte hypertrophy, leading to adipocyte dysfunction presenting with inflammation and insulin resistance [14,15]. In addition, adipocytes secrete adipokines such as adiponectin, monocyte chemoattractant protein-1, and tumour necrosis factor-α (TNF-α), which promote chronic vascular inflammation and destruction of the extracellular matrix, leading to AAA formation [10]. In addition to adipokines, oxidative stress also plays an important role in inducing the signalling pathway related to the progression of AAA [16,17]. However, the effects of hypoperfusion associated with low glucose- and serum-level induced inflammatory responses in adipocytes of the AAA wall are not fully understood. Low glucose and serum levels may act through multiple mechanisms to alter adipocyte function. The purpose of this study was to identify the effect of low glucose and serum deprivation conditions on the inflammation-related factors in adipocytes.

## Results

### Viability of 3T3-L1 cells under conditions of low glucose and serum levels

**Figure 1.**
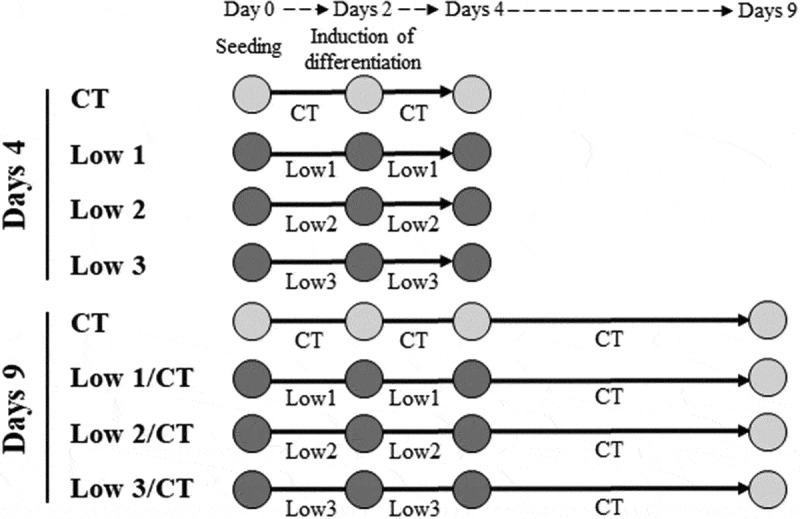
Experimental condition for days 4 and days 9

To determine the effect of low glucose and serum levels on cell viability, we cultured 3T3-L1 cells in different culture media: control (CT), 4.5 g/L glucose and 10% foetal bovine serum (FBS); Low 1, 1.0 g/L glucose and 10% FBS; Low 2, 1.0 g/L glucose and 5% FBS; Low 3, 1.0 g/L glucose and 1% FBS; Low 4, 4.5 g/L glucose and 1% FBS; Low 5, 4.5 g/L glucose and 0% FBS. The viability of 3T3-L1 cells without induction of differentiation was significantly decreased in Low 3, −4, and −5 conditions compared to that in CT medium (Figure S1(a)). There were very few viable cells in Low 5 condition; therefore, this condition was eliminated from the subsequent experiments. Next, we investigated the effect of different culture media on the viability of cells following induction of differentiation, as shown in (Figure 1). Cell viability was significantly decreased in Low 1, −2, and −3 conditions compared to that in CT (Figure S1(b)). However, viability of the cells was not significantly different between Low 1, −2, and −3 (Figure S1(b)) conditions. Further, the decreased cell viability was restored by returning the cells to normal culture medium (cell viability > 80%) (Figure S1(b)). Based on these results, we selected a Low 3 condition as the optimum low glucose and serum level model in this study to arrest the growth of 3T3-L1 adipocytes.

### Low glucose and serum level-induced intracellular production of reactive oxygen species (ROS), nitric oxide (NO) secretion, and inducible nitric oxide synthase (iNOS) expression in 3T3-L1 cells

**Figure 2.**
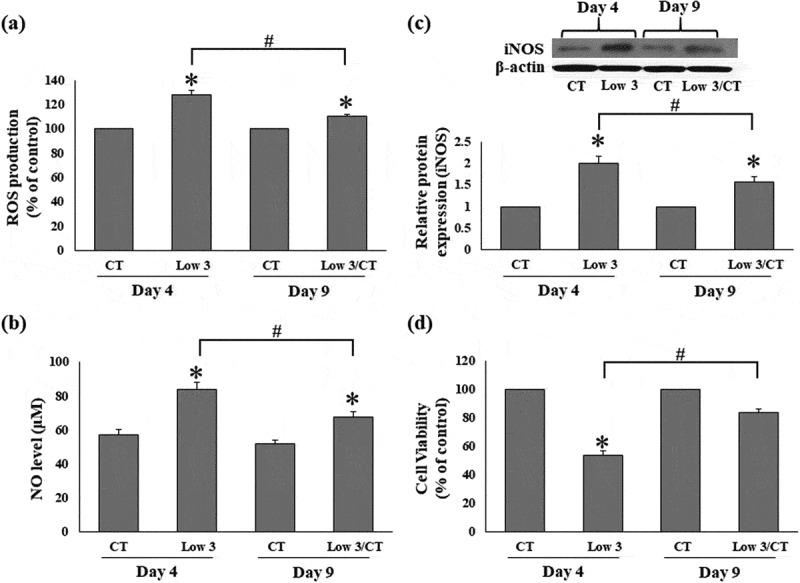
Low glucose and serum levels increases ROS and NO level and iNOS expression. (a) Intracellular ROS production, (b) NO level, (c) gel images and the relative protein level of iNOS and (d) the cell viability. Values are expressed as means ± S.E.M (n = 4). *P < 0.05 compared to the CT group of each time, #P < 0.05 compared to the Low 3

Next, we evaluated the effect of low glucose and serum levels on the generation of ROS and NO, and expression of iNOS. Intracellular ROS production by 3T3-L1 cells increased significantly to 128.31 ± 1.70% under Low 3 condition compared to that in CT condition on day 4, whereas it was significantly suppressed in Low 3/CT conditions on day 9 (Figure 2(a)). Compared to CT condition, NO levels and iNOS protein expression were significantly enhanced under Low 3 conditions on day 4 (Figure 2(b,c)). Under Low 3/CT conditions, NO secretion and iNOS protein expression were significantly decreased compared to that in the Low 3 condition on day 9 (Figure 2(b-c)). ROS and NO regulate cell viability, and the relative growth rates was concomitantly decreased in Low 3 condition when compared to that in the CT condition (day 4). However, the growth rate in Low3/CT condition returned to the normal level, as shown in Figure 2(d). These data suggest that an increase in NO and ROS levels may be related to the suppression of adipocyte proliferation.

### Low glucose and serum level-induced TNF-α and interleukin-1β (IL-1β) secretion in 3T3-L1 cells

**Figure 3.**
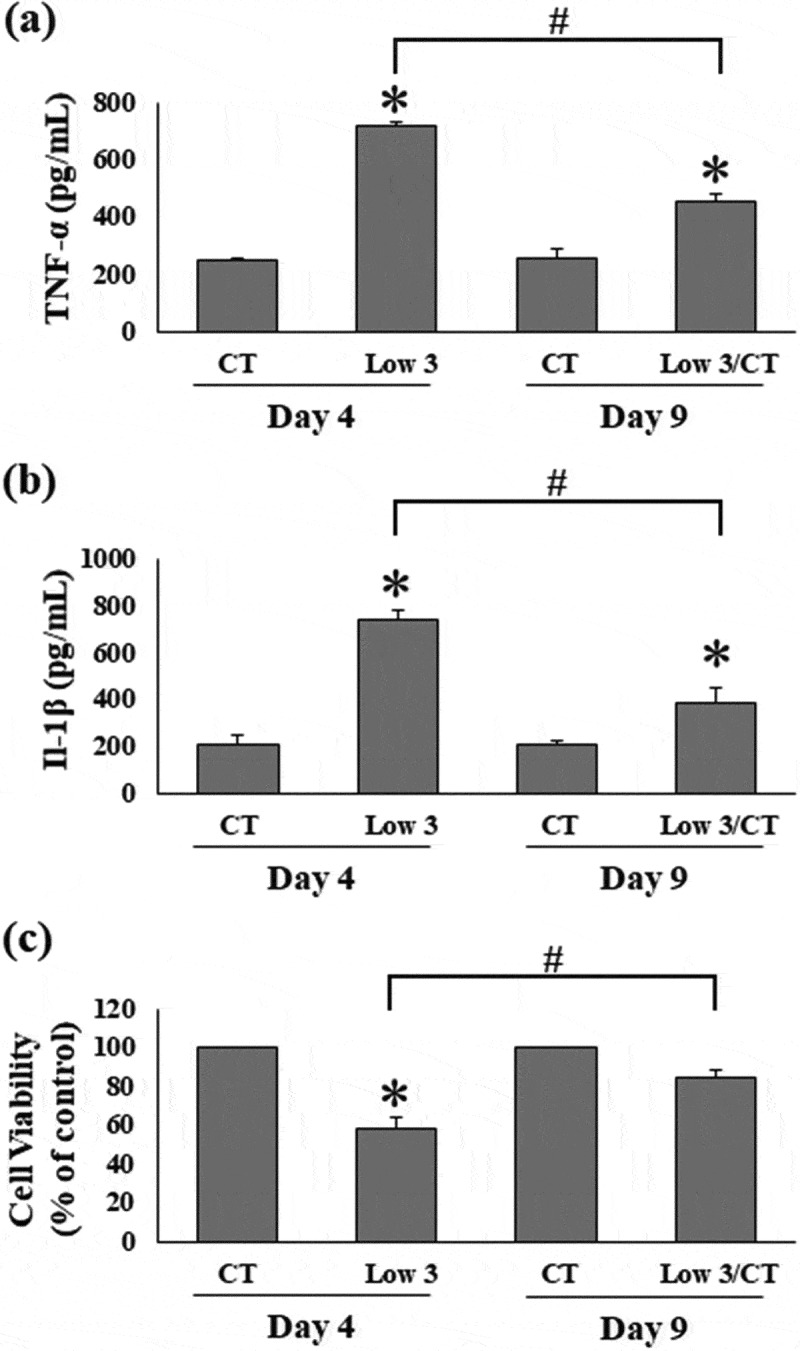
Low glucose and serum levels increases TNF-α and IL-1β. (a) TNF-α level, (b) IL-1β level and (c) cell viability are shown. Values are expressed as means ± S.E.M (n = 4). *P < 0.05 compared to the CT group of each time, #*P* < 0.05 compared to the Low 3

Next, we assessed the effect of low glucose and serum levels on TNF-α and IL-1β secretion by 3T3-L1 cells. Compared to the CT condition, levels of TNF-α and IL-1β secreted in the culture media were significantly increased under Low 3 conditions on day 4 (Figure 3(a,b)). Compared to the Low 3 condition, TNF-α and IL-1β levels were decreased under Low 3/CT conditions on day 9 (Figure 3(a,b)). Cell viability was used to determine the role of cell number on cytokine levels, and our results showed that the cell viability decreased to 57.78 ± 5.76% in Low 3 condition when compared to that in CT on day 4 (Figure 3(c)), in contrast to the levels of TNF-α and IL-1β (Figure 3(a,b)). Viability of cells in Low 3/CT condition returned to the normal level (84.70% ± 3.31%), as shown in Figure 3(c), consistent with the decrease in TNF-α and IL-1β levels (Figure 3(a,b)). The present findings imply that high levels of proinflammatory cytokines regulate the viability of adipocytes.

### Low glucose and serum level-induced matrix metalloproteinase (MMP)-9 expression and activity in 3T3-L1 cells

**Figure 4.**
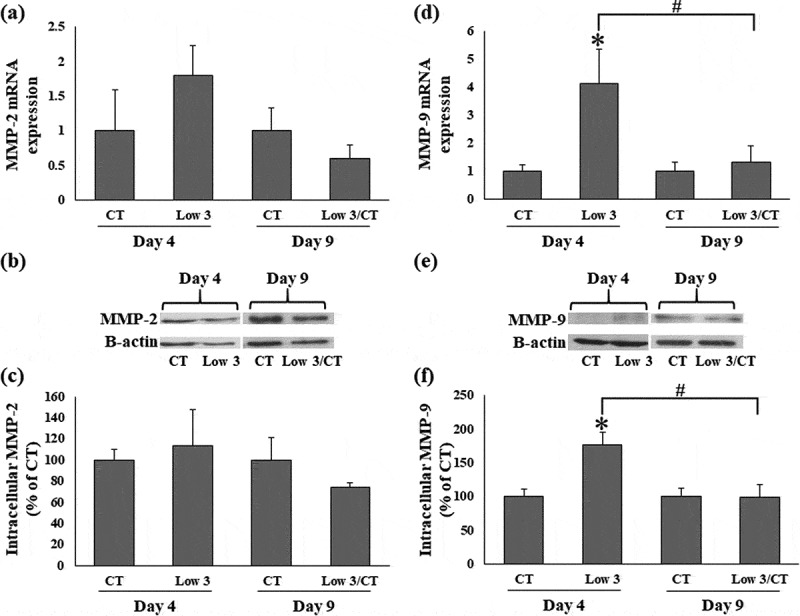
Effects of low glucose and serum levels culture on MMPs expression. (a) Relative mRNA expression of MMP-2, (b) gel images showing protein expression of MMP-2 and (c) relative protein expression of MMP-2. (d) Relative mRNA expression of MMP-9, (e) gel images showing protein expression of MMP-9 and (f) relative protein expression of MMP-9. Data are represented as mean ± S.E.M (n = 4). *P < 0.05 compared to the CT group of each time, #*P* < 0.05 compared to the Low 3

**Figure 5.**
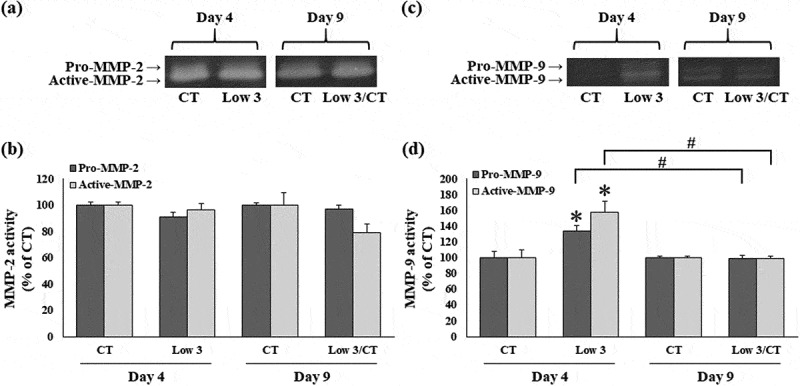
Effects of low glucose and serum levels culture on MMPs activity. (a) Gel images showing MMP-2. (b) Relative activity of MMP-2. (c) Gel images showing MMP-9. (d) Relative activity of MMP-9. Data are represented as mean ± S.E.M (n = 4). *P < 0.05 compared to the CT group of each time, #*P* < 0.05 compared to the Low 3

To examine the effects of low glucose and serum levels on the expression of MMP-2 and MMP-9, mRNA and protein levels of MMP-2 and MMP-9 were determined by real-time PCR and western blotting, respectively (Figures 4 and 5). MMP-2 mRNA and protein expression in 3T3-L1 cells was not significantly different under Low 3 conditions compared to that in CT condition on day 4 (Figure 4(a-c)). Furthermore, under Low 3/CT conditions, MMP-2 mRNA and protein levels were not significantly different compared to those in the Low 3 condition on day 9 (Figure 4(a-c)). Compared to the CT condition, the MMP-9 mRNA and protein levels in 3T3-L1 cells were significantly enhanced in the Low 3 condition on day 4 (Figure 4(f)). However, compared to the Low 3 condition, MMP-9 mRNA and protein levels were significantly decreased in the Low 3/CT condition on day 9 (Figure 4(f)).

The activity of MMPs in the conditioned medium was determined using gelatin zymography (Figure 5). The activities of pro-MMP-2 and active-MMP-2 in 3T3-L1 cells growing in culture medium under Low 3 conditions were compared to that of cells under CT conditions on day 4 (Figure 5(a,b)). Furthermore, under Low 3/CT conditions, the activities of pro-MMP-2 and active-MMP-2 were not significantly different from those under Low 3 conditions on day 9 (Figure 5(a,b)). Compared to CT condition, the activities of pro-MMP-9 and active-MMP-9 were significantly increased under Low 3 conditions on day 4 (Figure 5(c,d)). However, compared to the Low 3 condition, the activities of pro-MMP-9 and active-MMP-9 were significantly decreased under Low 3/CT conditions on day 9 (Figure 5(c,d)).

### Low glucose- and serum level-induced the activation of extracellular signal-regulated protein kinase (ERK1/2), c-Jun N-terminal kinase (JNK), and p38 mitogen activated protein kinase (MAPK) signalling pathway in 3T3-L1 cells

**Figure 6.**
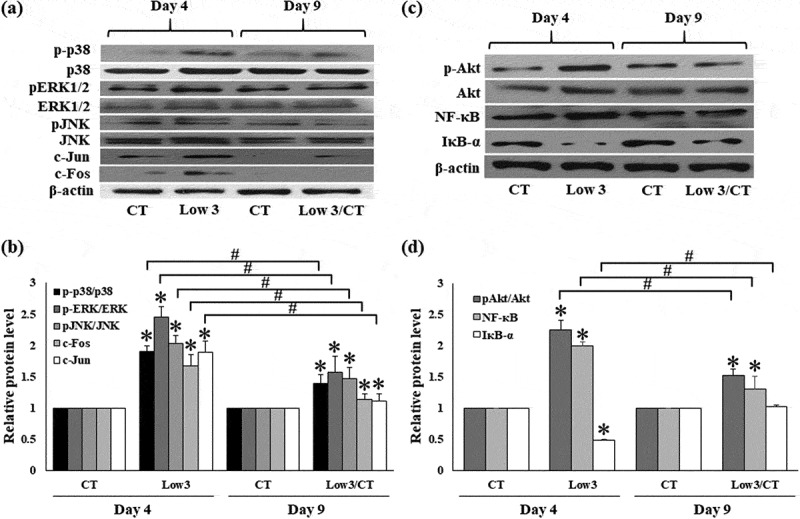
Effects of low glucose and serum levels culture on ERK1/2, JNK, p38 MAPKs, c-Fos, c-Jun, Akt, NF-кB and IкB-α expressions. (a) Gel images showing protein expression of ERK1/2, JNK, p38 MAPKs, c-Fos and c-Jun (b) the relative protein level of ERK1/2, JNK, p38 MAPKs, c-Fos and c-Jun (c) gel images showing protein expression of Akt, NF-кB and IкB-α and (d) the relative protein level of Akt, NF-кB and IкB-α. Values are expressed as means ± S.E.M (n = 4). *P < 0.05 compared to the CT group of each time, #*P* < 0.05 compared to the Low 3

Next we examined whether MAPK activation was involved in the oxidative stress and inflammation induced by low glucose and serum levels. Low glucose and serum levels significantly stimulated p38, ERK1/2, and JNK phosphorylation compared to CT in 3T3-L1 cells after 4 days (Figure 6(a,b)). Moreover, western blot analysis using adipocyte nuclear extracts revealed that Fos and Jun proteins were induced under Low 3 conditions (Figure 6(a,b)). Consistent with the decrease in Fos and Jun proteins, the phosphorylation of p38, ERK1/2, and JNK was significantly decreased under Low 3/CT conditions on day 9 compared to that under Low 3 condition (Figure 6(a,b)). These results indicated that low levels of glucose and serum enhanced the inflammatory response in the early phase of adipogenesis, whereas normal levels of glucose and serum markedly decreased the inflammatory response by suppressing the MAPK signalling pathway in adipocytes.

### Low glucose- and serum level-induced the activation of Akt and nuclear factor (NF)-кB signalling pathway in 3T3-L1 cells

The expression of pAkt/Akt was significantly increased in cells under low glucose and serum levels compared to that under CT condition, and the phosphorylation of Akt was downregulated in Low 3/CT condition compared to that in Low 3 condition (Figure 6(c,d)). NF-κB activation is necessary for the development of inflammation, and for the production of reactive species, and inflammatory cytokines. Thus, we explored the effect of nutrient stress on NF-κB and IкB-α expression in 3T3-L1 cells. Nutrient stress significantly increased p65 NF-κB and decreased IкB-α protein expression compared to that in CT in 3T3-L1 adipocyte-differentiated cells on day 4 (Figure 6(c,d)). Interestingly, the expression of p65 NF-κB decreased significantly whereas that of IкB-α increased significantly in Low 3/CT conditions on day 9 when compared to that in Low 3 conditions (Figure 6(c,d)).

## Discussion

A previous study found that hypoperfusion of adventitial VV due to stenosis causes the onset of AAA by inducing ectopic adipocytes in the AAA wall [4,5]. Abnormal adipocytes in the AAA wall are reportedly associated with AAA development and rupture [6,8–10,13,18]. In this study, we examined the effect of nutrient stress on changes in the expression of inflammatory factors in 3T3-L1 cells to identify the factors associated with hypoperfusion and AAA.

Oxidative stress is associated with multiple mechanisms in AAA, including vascular inflammation and increased metalloproteinase activity [19]. ROS and reactive nitrogen species have been reported to be upregulated in both human tissue and animal models [17,20,21], and are associated with the pathogenesis of AAA. In this study we found increased production of ROS and NO in 3T3-L1 cells cultured in low-nutrient medium. Increase in NO levels was a result of the upregulation of iNOS expression. These results are consistent with the previous finding that low glucose and serum levels cause endoplasmic reticulum stress and increase the production of ROS by mitochondria [22,23]. Significant increases in ROS and NO generation, and iNOS expression have been observed in human AAA tissue [24]. Interestingly, we found that the levels of ROS, NO production, and iNOS protein expression were significantly restored by normal nutritional conditions. Our data indicate that major increases in ROS and NO production are associated with the depletion of glucose and serum levels in the medium, that also induces adipocyte stress. Both of these parameters can be maintained at a low level by maintaining normal glucose and serum levels.

Previous studies have shown that ROS induces the expression of matrix-degrading enzymes, such as MMP-2 and MMP-9, and proinflammatory cytokines such as TNF-α and IL-1β [19,24]. In addition, adipocytes produce MMP-2, MMP-9, TNF-α, and IL-1β [25,26]. This study showed that 3T3-L1 cells cultured in medium with depletion of glucose and serum levels showed increased production of TNF-α and IL-1β, and increased expression and activation of MMP-9. Notably, our results indicated that the depletion of glucose and serum levels significantly induced inflammation in adipocytes, which was consistent with a previous study illustrating the effect of low glucose on the proinflammatory cytokine IL-1β level in monocytes [27] and hypoglycaemia induced increase in proinflammatory cytokines (TNFα, IL-1β, IL-6, and IL-8) and oxidative stress in healthy individuals [28]. These results suggest that VV stenosis causes low vascular glucose and serum levels and may shift adipocytes into an inflammatory stage through an increase in oxidative stress, proinflammatory cytokine production, and promotion of fibre degradation through the upregulation of MMPs in the AAA wall. Normal nutrient supply to the vascular wall may be important in preventing hypoperfusion-induced AAA. However, MMP-2 did not change under our experimental conditions, suggesting that other unidentified mechanisms regulate MMP-2 expression under our experimental conditions.

NF-кB is closely associated with the regulation of inflammation and immune responses in the physiological or pathophysiological processes of AAA [29]. Several stimuli, such as angiotensin II, ROS, and MMP-9, activate NF-κB signalling in AAA diseases [30–32]. In this study, depletion of glucose and serum induced activation of NF-кB and inhibition of IкB-α in 3T3-L1 adipocytes, consistent with the results of Kohno et al. [33], who reported that serum starvation stress induced NF-kB activation by exclusion of the negative factor of serum. In addition, both low and high glucose concentrations stimulate NF-кB to contribute to the inflammatory response [34]. Based on our findings, induction of MMP-9, IL-1β, and TNF-α may be due to the stimulation of NF-кB and suppression of IкB-α in the adipocytes.

Increased levels of phosphorylated ERK and JNK have been observed in both human and rodent AAA tissues [35–37]. MAPKs are recognized as pivotal proteins in different signalling events mediated through phosphorylation cascades, and MAPK signalling also controls numerous intracellular events in adipocytes, especially inflammation [38,39]. Upregulation of ROS, NO, proinflammatory cytokines, and cellular stress is known to trigger MAPK activation, including ERK, p38, and JNK that are implicated in the initiation of preadipocyte differentiation, adipocyte hypertrophy, and inflammation [38,39]. We tested the involvement of serum components and glucose in inflammatory adipocytes induced changes in the MAPK pathway by creating nutrient stress conditions. Our results showed that glucose and serum starvation induced nutrient stress and significantly increased the activation of ERK1/2, JNK, and p38 MAPK, suggesting that this pathway mediates the increased level of inflammatory factors in 3T3-L1 cells, which is consistent with the results of previous studies performed in various cell types including adipocytes [40], macrophages, and glioblastoma cells [41]. Moreover, the activation of Jun and Fos (subunits of the AP-1 transcriptional factor), targets of the ERK/JNK signalling pathway, was also found to be upregulated during glucose and serum stress. MAPK has been reported to be involved in regulating adipocyte inflammation, with subsequent activation of NF-κB [39]. This demonstrates that the ERK1/2, p38, and JNK signalling pathways with subsequent activation of NF-κB play an important role in glucose and serum deprivation-induced inflammation in adipocytes by regulating the generation of NO, ROS, and proinflammatory cytokines.

Recently, Akt signalling was reported to be increased during AAA development, and suppression of Akt inhibited AAA formation [29]. Our results showed that upon returning to normal nutrient medium, 3T3-L1 adipocyte-differentiated cells significantly suppressed the glucose- and serum deprivation-induced increase in phosphorylated-Akt expression. These data suggest that glucose- and serum deprivation-induced inflammatory changes in 3T3-L1 adipocyte-differentiated cells are associated with the activation of the Akt pathway.

In conclusion, glucose and serum deprivation may induce inflammation in adipose tissue through the activation of MAPKs, Akt, and NF-кB signalling pathways. Nutritional supplementation may attenuate glucose- and serum deprivation-induced inflammation in the AAA wall. A limitation of this study is the lack of information concerning the interactional response of different cell populations in the AAA wall as we used only one type of cell culture to determine the effect of low glucose and serum levels on adipocytes. Further investigations are required to clarify the relationship between our experimental conditions and inflammation in hypoperfused AAA wall.

## Materials and methods

### Cell culture and adipocyte differentiation

Mouse 3T3-L1 adipocytes were purchased from the American Type Culture Collection (ATCC, Manassas, VA, USA). The cells were plated in a 24-well plate and maintained in Dulbecco’s modified Eagle’s medium (DMEM; Nissui Pharmaceutical Co., Tokyo, Japan) supplemented with 10% foetal bovine serum (FBS; Gibco™, Life Technologies, Rockville, MD, USA), 1% penicillin-streptomycin, 2 mM L-glutamic acid, 100 mM pyruvic acid, 4.5 g/L glucose, and 7.5% sodium carbonate at 37°C under an atmosphere of 5% CO_2_. Induction of adipocyte differentiation was induced using Adipo Inducer Reagent (for animal cells, Takara Bio Inc., Shiga, Japan) containing insulin, dexamethasone, and 3-isobutyl-1-methylxanthine. Cell viability assay was performed using the CellTiter96 Aqueous One Solution Cell Proliferation Assay kit (Promega, Fitchburg, WI, USA).

To estimate the effect of nutrient stress, we cultured cells in the following condition mediums for 4 days: control, 4.5 g/L glucose and 10% FBS; Low 1, 1.0 g/L glucose and 10% FBS; Low 2, 1.0 g/L glucose and 5% FBS; Low 3, 1.0 g/L glucose and 1% FBS; Low 4, 4.5 g/L glucose and 1% FBS; Low 5, 4.5 g/L glucose and 0% FBS. To estimate the effect of restoration by returning normal nutritional condition, we cultured cells for 9 days in Low/CT groups.

### Intracellular ROS assay

The oxidation of 2ʹ,7ʹ-dichlorofluorescin diacetate (DCFH-DA; Sigma-Aldrich, St. Louis, MO, USA) was used to determine intracellular ROS production in the cells. Cells were treated with DCFH-DA (50 µM) for 1 h in the dark. The intensity of the fluorescent product, dichlorofluorescein (DCF), was determined at 485 (excitation wavelength) and 530 nm (emission wavelength) using a fluorescence microplate reader (Bio-Tex Instruments, Inc., VT, USA).

### NO assay

Equal volumes of Griess reagent and culture media were mixed and incubated in the dark for 10 min at room temperature. The optical density of the nitrite-containing samples was measured using a microplate reader (Bio-Tex Instruments) at 540 nm. A standard calibration curve was generated using sodium nitrite.

### TNF-α and IL-1β assay

The level of TNF-α and IL-1β was analysed using an enzyme-linked immunosorbent assay (ELISA; Merck Millipore, Darmstadt, Germany).

### Gelatin zymography

The MMPs were separated on a 7.5% polyacrylamide resolving gel containing 2.5 mg/mL gelatin, and the gel was incubated for 30 min at room temperature in a 2.5% Triton X-100 solution and incubated at 37°C for 18 h in 50 mM Tris–HCl buffer, pH 8.0, containing 10 mM CaCl_2_. The gels stained with 0.1% amido black 10B (Wako Pure Chemical Industries, Osaka, Japan).

### Real-time PCR

Total RNA was isolated using Sepasol-RNA II Super (Nacalai Tesque, Kyoto, Japan) and TURBO DNA-free^TM^ Kit (Thermo Fisher Scientific, MA, USA). cDNA was synthesized using PrimeScript^TM^ II 1^st^ strand cDNA Synthesis Kit (Takara Bio. Inc.). Real-time PCR was conducted using SYBR Premix Ex Taq II (Takara Bio. Inc.). Primers used for the real-time PCR experiments are shown in Table S1. The amplification conditions used were 5 s at 95°C and 30 s at 65°C for 40 cycles. *β-actin* served as the internal control.

### Sodium dodecyl sulphate polyacrylamide gel electrophoresis (SDS-PAGE) and western blot analysis

Protein extracts were boiled for 5 min in SDS sample buffer, separated using SDS-PAGE, and transferred onto polyvinylidene difluoride membranes (Immobilon™-P, MerckMillipore, Billerica, MA, USA). The membranes were probed with the following primary antibodies: rabbit anti-MMP-2 (1:1000; Novus Biologicals, Littleton, CO, USA), mouse anti-MMP-9 (1:300; Abcam, Tokyo, Japan), Akt (1:1000; Santa Cruz, California, USA), pAkt (1:1000; Santa Cruz), p65 NF-κB (1:1000; Santa Cruz), IкB-α (1:1000; Santa Cruz), ERK1/2 (1:1000; Abcam, Cambridge, UK), pERK1/2 (1:1000; Abcam), p38 (1:1000; Abcam), p-p38 (1:1000; Abcam), JNK (1:1000; Abcam), pJNK (1:1000; Abcam), c-Fos (1:500; Santa Cruz), c-Jun (1:500; Santa Cruz), iNOS (1:1000; Santa Cruz), and mouse anti-β-actin (1:10,000; Merck). All immunoreactive proteins were detected using ECL™ Western Blotting Detection Reagent (GE Healthcare, Buckinghamshire, UK).

### Statistical analysis

Statistical differences were determined using the Tukey-Kramer test. Differences were considered significant at *P* < 0.05. Statistical analyses were performed using Stat View 5.0 software (SAS Institute, Tokyo, Japan).

## Supplementary Material

Supplemental Material
