# Supplementary material for: Low glucose and serum levels cause an increased inflammatory factor in 3T3-L1 cell through Akt, MAPKs and NF-кB activation
Source: Adipocyte. 2021 Apr 25;10(1):232–41. doi: 10.1080/21623945.2021.1914420 (PMC8078669; doi:10.1080/21623945.2021.1914420)
Supplement: Supplemental Material [file KADI_A_1914420_SM2571.pdf]

(a)

Group	Medium: Glucose (g/L)	Medium: FBS (%)	Viable cell count (×10^5 cells/mL)
CT	4.5	10	3.3 ± 0.23
Low 1	1	10	3.3 ± 0.35
Low 2	1	5	3.0 ± 0.61
Low 3	1	1	1.2 ± 0.40*
Low 4	4.5	1	2.0 ± 0.03*
Low 5	4.5	0	0.1 ± 0.04*

(b)

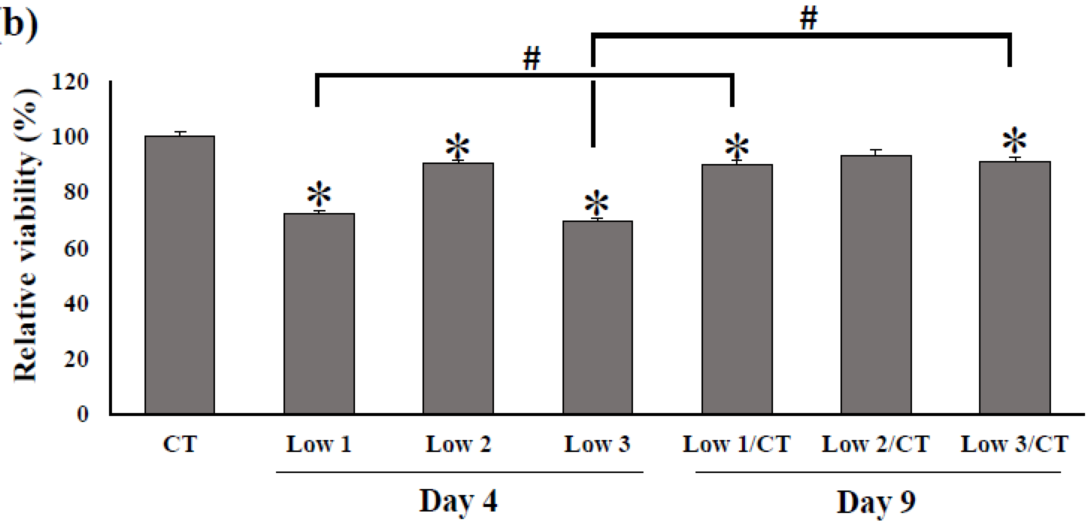

Figure S1. Viable cell count and cell viability under low glucose and serum levels. (a) Composition of medium and viable cell count (×10^5 cells/mL). Control (CT), 4.5 g/L glucose and 10% FBS; Low 1, 1.0 g/L glucose and 10% FBS; Low 2, 1.0 g/L glucose and 5% FBS; Low 3, 1.0 g/L glucose and 1% FBS; Low 4, 4.5 g/L glucose and 1% FBS; Low 5, 4.5 g/L glucose and 0% FBS. (b) Relative viability of 3T3-L1 cells on days 4 and 9. Data are represented as mean ± S.E.M (n = 4). *P<0.05 compared to the CT group. #P<0.05 between Low1 and Low1/CT, and between Low3 and Low3/CT.

Table S1. Primers used for real-time PCR experiments.

Gene		Primer sequence
MMP-2	FWD	5'-ATTGAAGCTGGAGAACCAA-3'
	REV	5'-CTTTGGGCACAAAAAGAAGC-3'
MMP-9	FWD	5'-TTCGCGTGGATAAGGAGTTC-3'
	REV	5'-CGGTTGAAGCAAAGAAGGAG-3'
β-actin	FWD	5'-CCAACCGTGAAAAGATGACC-3'
	REV	5'-CCAGAGGCATACAGGGACAG-3'
